# Molecular fingerprinting of bovine mastitis-associated *Staphylococcus aureus* isolates from India

**DOI:** 10.1038/s41598-021-94760-x

**Published:** 2021-07-27

**Authors:** Madhavi Annamanedi, P. Sheela, Srinivasaiah Sundareshan, Shrikrishna Isloor, Priya Gupta, Pagala Jasmeen, Madhuranjana Gargi, Sathi Mallick, Nagendra R. Hegde

**Affiliations:** 1 National Institute of Animal Biotechnology, Opposite Journalist Colony, Extended Q City Road, Near Gowlidoddi, Gachibowli, Hyderabad, 500032 India; 2 Department of Microbiology, Veterinary College, Karnataka Veterinary Animal and Fisheries Sciences University, Vinobanagar, Shivamogga, Karnataka 577204 India; 3 Department of Microbiology, Veterinary College, Karnataka Veterinary Animal and Fisheries Sciences University, Hebbal, Bangalore, 560024 India

**Keywords:** Infectious-disease epidemiology, Bacterial infection, Bacterial pathogenesis

## Abstract

*Staphylococcus aureus* is a major etiological agent of clinical and subclinical bovine mastitis. Owing to the mostly backyard dairy practices, we hypothesized that genetic diversity among mastitis-associated *S. aureus* from India would be high, and investigated 166 isolates obtained mostly from the Southern State of Karnataka, but also from a few other states. The results revealed (a) 8 to 13 fragments in pulsed-field gel electrophoresis (PFGE), forming 31 distinct patterns, and (b) 34 *spa* types, of which three (t17680, t18314, and t18320) were newly identified. Multi-locus sequencing typing (MLST) identified 39 sequence types (STs), with ST2454 (34.4%) and ST2459 (24%) being the most commonly represented, which clustered to clonal complexes (CC) CC9 and CC97, respectively; 12 STs were newly identified. Thirty-four (20.5%) of the 166 isolates displayed oxacillin resistance. On the other hand, whereas none were *mec*C^+^, 44 (26.5%) isolates were *mec*A^+^, with a predominance of SCC*mec*IVb (26/32 isolates, others being untypeable); 24 isolates (14.46%) were oxacillin-susceptible methicillin-resistant *S. aureus* (OS-MRSA; *mec*A^+^ but OS). Integrated analysis revealed that CC9-ST2454- and CC97-ST2459-SCC*mec*IVb were the predominant MRSA, although the distribution of CC9 and CC97 was similar between methicillin-resistant and -susceptible isolates. By PCR, 56.25%, 28.75% and 47.5% of the 166 isolates were positive for *hlg*, *tsst* and *pvl* genes, respectively*.* Our results, for the first time describe the application of a combination of various molecular methods to bovine mastitis-associated *S. aureus* isolates from India, corroborate the worldwide distribution of CC97 and CC9, and suggest pathogenic potential of the isolates.

## Introduction

Infections and dynamics of disease caused by the same pathogen differ from host to host and from one geographical location to another. Some clonal lineages of a particular pathogen tend to be specific to a host, and others appear to transmit easily between hosts. To gain knowledge about similarities among strains, sources of infection, modes of transmission and carriage of virulence and resistance genes, one can employ molecular typing methods which can reveal genetic diversity of the isolates of a particular pathogen.

*Staphylococcus aureus* is found associated with a variety of human infections, with some evidence for overlap in the distribution of certain clonal populations between humans and animals^1–4^. The organism is known to produce a number of virulence factors such as toxins and antibiotic resistance mediators which facilitate its survival in the host as well as in the environment^5,6^. In addition, methicillin-resistant *S. aureus* (MRSA), which encode multiple antimicrobial resistance genes, are a major problem in human medicine^7^. Bovine mastitis is a very common livestock production-related disease, and *S. aureus* is the major contributor, resulting in a range of manifestations, including a large proportion of subclinical and chronic cases^8,9^. Isolates from bovine mastitis have been shown to produce various types of hemolysins and enterotoxins, as well as Panton-Valentine leucocidin (PVL) and toxic shock syndrome toxin (TSST), although the proportions of the isolates expressing each type of these toxins vary widely^10–12^. Although early studies suggested much lower prevalence of MRSA among bovine mastitis-associated *S. aureus* compared to those isolated from human infections, recent studies have shown trends towards higher prevalence^13–26^. This has led to focus towards the longitudinal spread and sharing of particular MRSA clones and their related strains among and between animals and humans, in order to understand the consequence of animal-human interface in infections and diseases caused by MRSA.

Multiple genetic types of bovine mastitis-associated *S. aureus* exist worldwide. Studying the origin, clonal populations, genetic variants and virulence determinants of *S. aureus* is important for the understanding of epidemiology, ecology and host adaptation, zoonotic potential, association with disease, and possibly to predict clinical outcome and control of disease. Typing methods for *S. aureus* include pulsed-field gel electrophoresis (PFGE), staphylococcal protein A (*spa*) typing and multi-locus sequence typing (MLST)^7^. PFGE provides reliable epidemiological comparisons among isolates, and is based on macro-restriction fragment polymorphism of genomic DNA. *Spa* typing analyses sequence repeats within the X region of the *spa* gene^27^. MLST analyses the sequence polymorphism of seven housekeeping genes of each isolate; MLST facilitates comparisons of population structures with high levels of discrimination and reproducibility^28^. These typing methods provide tools to examine the epidemiological picture in terms of diversity and clonality of *S. aureus*.

Most of the studies investigating genotypes of mastitis-associated *S. aureus* have been from large dairy herds, where it may not be surprising to see high level of clonality. Indian dairy industry is majorly based on pooling practices where individual holders keeping a few animals contribute to pooling of milk through dairy cooperatives. Because of the scattering of animals, we hypothesized that mastitis-associated *S. aureus* strains from India would be quite diverse. Very few studies have examined genotypes of bovine mastitis-associated *S. aureus* in India. In one study, where 39 MRSA isolates from three different states (Andhra Pradesh, Karnataka, Telangana) were investigated, *spa* types t037 and t267 and sequence types (ST) 72, ST245 and ST239 were reported to be predominating despite some diversity^29^. In another study with 100 isolates, *spa* types t359, t7867 and t3841 were found to be predominant in Karnataka^30^. In a third study, where 16 isolates were identified as MRSA, 50% of the isoltes belonged to ST1687^31^. Another recent report from West Bengal described the existence of t304 and t6267 types among nine MRSA isolates^32^. In this study, we analysed 166 isolates of *S. aureus* obtained from bovine mastitis cases from six different states of India using *spa*, MLST and PFGE, and found the existence of limited CCs with diversity at genotype level.

## Results

**Figure 1:**
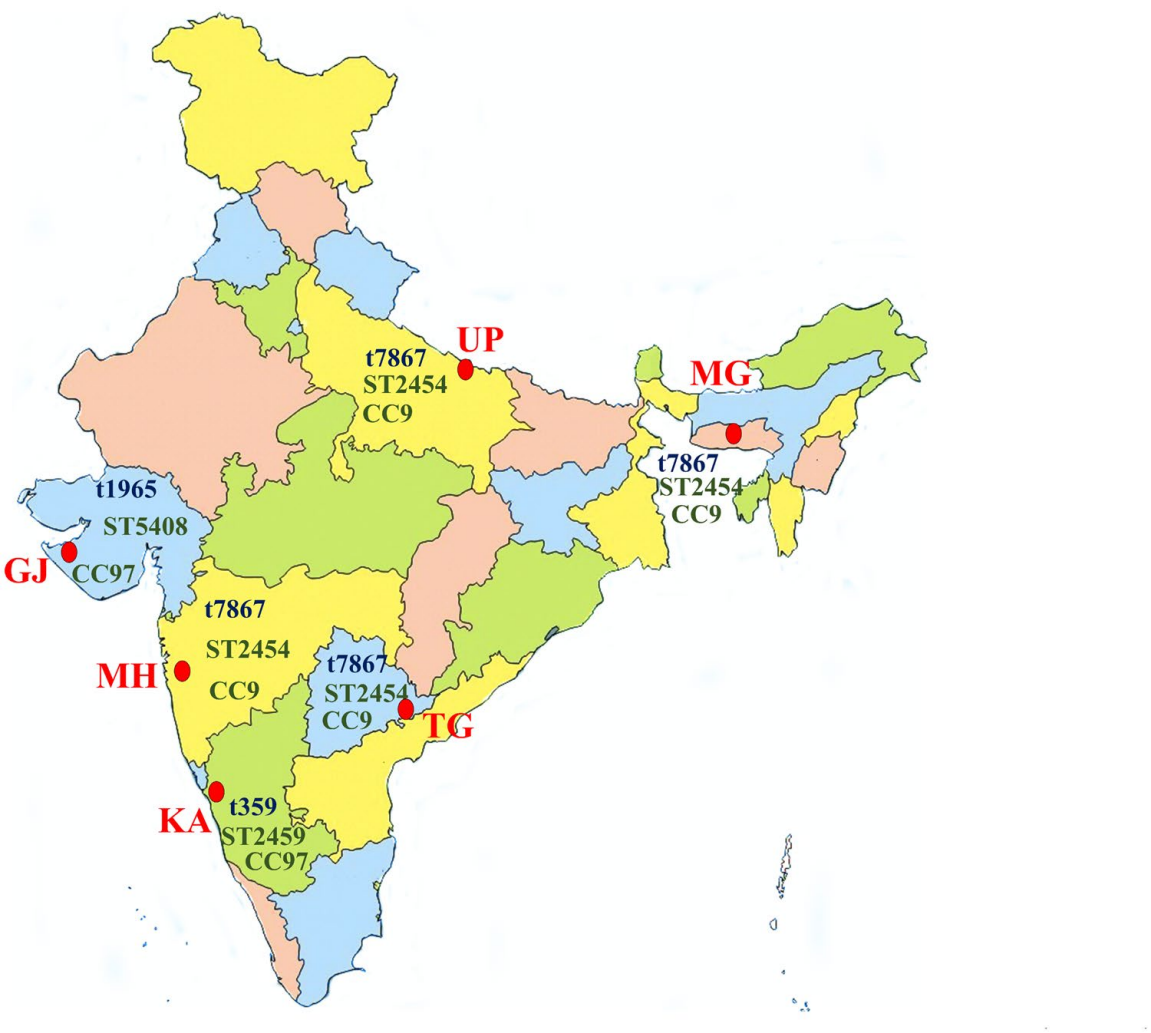
Map of India showing location of the six states from where bovine *S. aureus* isolates were obtained. For each state, the predominant *spa* type, sequence type and clonal complex are provided. GJ = Gujarat, KA = Karnataka, MH = Maharashtra, MG = Meghalaya, TG = Telangana, UP = Uttar Pradesh. The map was modified from one downloaded from the URL https://d-maps.com/carte.php?&num_car=24867&lang=en; from where up to 10 maps can be used per publication for free, as per their terms and conditions of use.

In this study, 166 bovine mastitis-associated *S. aureus* isolates from six different states of India were studied. The isolates included 98 from Karnataka, 19 each from Gujarat and Telangana, 12 from Uttar Pradesh and 5 from Maharashtra (Fig. 1).

**Figure 2:**
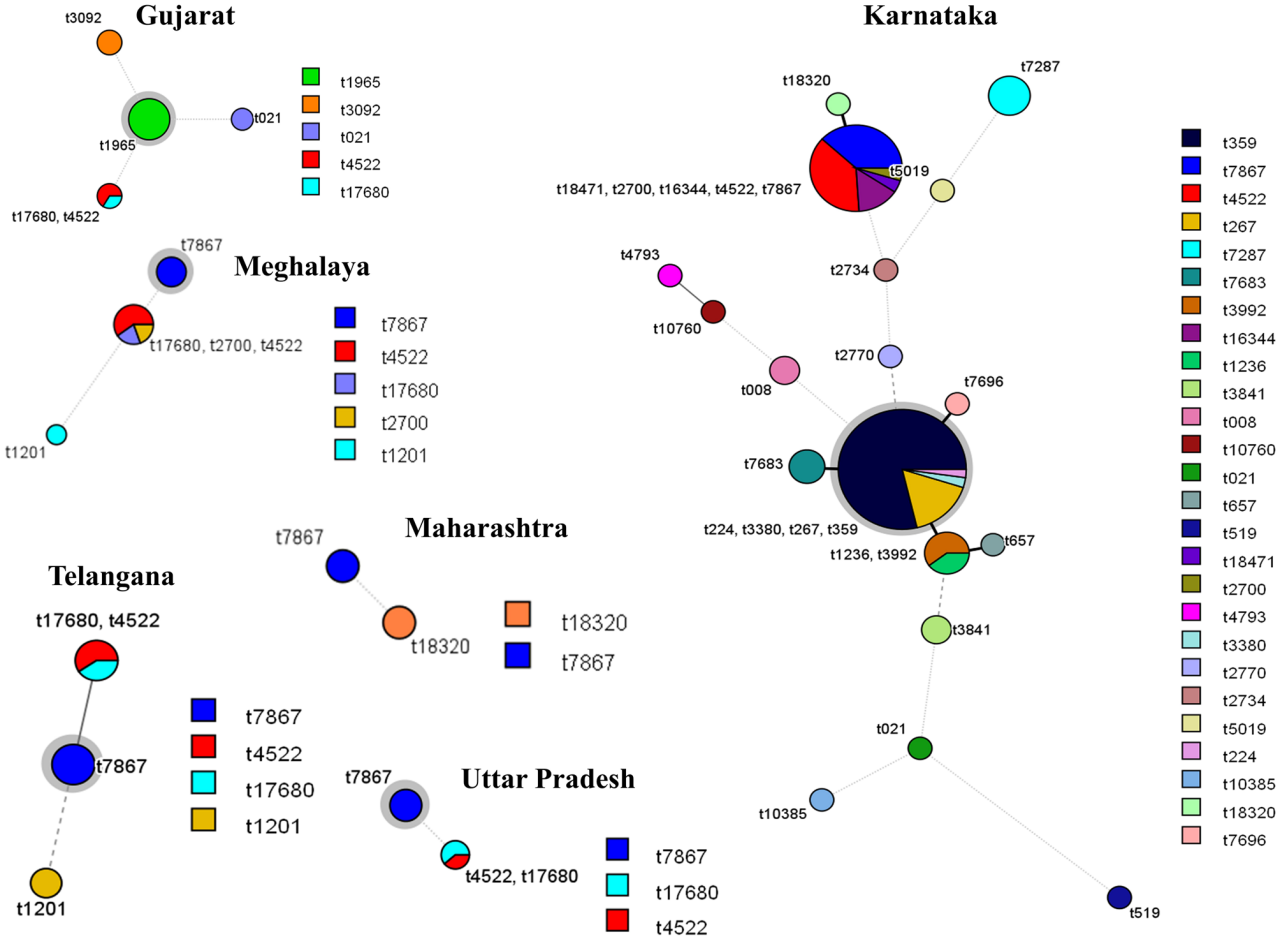
Minimum spanning trees showing *spa* types in each of the states. Each circle with different color represents *spa* type and size of the circle indicates the number of isolates for a particular *spa* type. Major *spa* type in each state is highlighted by a grey halo around the circle. The branch thickness (dotted lines, dashed lines and increasing thickness of solid lines in that order) indicates inverse of the distance between the types.

By *spa* typing, only 145 isolates were typeable and belonged to 34 different *spa* types. The predominant types among isolates from Karnataka and Gujarat were t359 (31/98, 30.3%) and t1965 (8/19, 42.1%), respectively. On the other hand, t7867 was the most frequent type in Uttar Pradesh (6/12, 50%), Telangana (2/5, 40%) and Meghalaya (6/19, 31.5%) (Fig. 2). Three new types (t17680, t18314 and t18320) were identified in this study.

**Figure 3:**
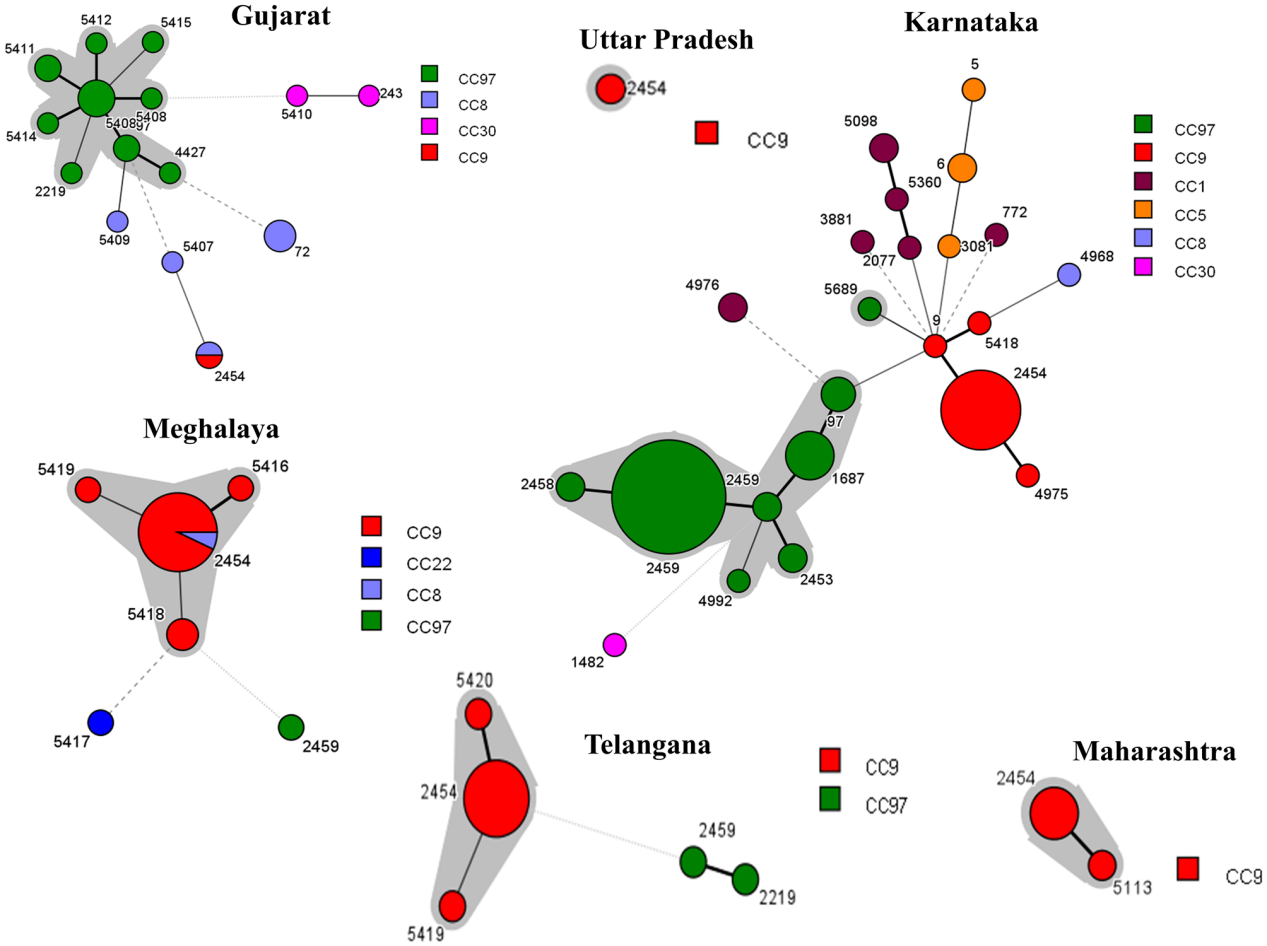
Minimum-spanning tree of MLST data from 166 *S. aureus* isolates in each state. Each circle with different color represents a different sequence type (ST) and the size of the circle is directly proportional to the number of isolates for a particular ST. Major clonal complex (CC) in each state is highlighted by a grey halo around the circle. The branch thickness (dotted lines, dashed lines and increasing thickness of solid lines in that order) indicates inverse of the distance between the STs.

By MLST, 39 sequence types (STs) were identified, with ST2454 (57/166, 34.4%) and ST2459 (40/166, 24%) being the most commonly represented. The most frequent STs from Karnataka and Gujarat were ST2459 (38/98, 38.7%) and ST5408 (4/19, 21%), respectively, whereas ST2454 predominated in Uttar Pradesh (12/12, 100%), Telangana (9/13, 69.2%), Meghalaya (13/19, 68.4%), and Maharashtra (4/9, 44.4%). In this study, 12 STs (ST5407, ST5408, ST5410, ST5411, ST5413, ST5414, ST5415, ST5416, ST5418, ST5419, ST5420 and ST5689) were newly identified. The allelic profiles for all the isolates are shown in Supplementary Table S2 and the minimum spanning tree showing the relatedness among the different STs is shown in Fig. 3.

The 39 STs could be grouped into seven CCs: CC1, CC5, CC8, CC9, CC30 and CC97, with CC97 and CC9 being predominant (Fig. 3). The dominant cluster from Gujarat (11/19, 57.8%) and Karnataka (53/98, 54%) was CC97, whereas CC9 dominated in Uttar Pradesh (12/12, 100%), Maharashtra (5/5, 100%), Meghalaya (17/19, 89.4%) and Telangana (11/13, 84.6%). Isolates belonging to CC1, CC5, CC8 and CC30 (20/166, 12%) were only identified from the states of Gujrat (6/20, 30%) and Karnataka (14/20, 70%).

**Figure 4:**
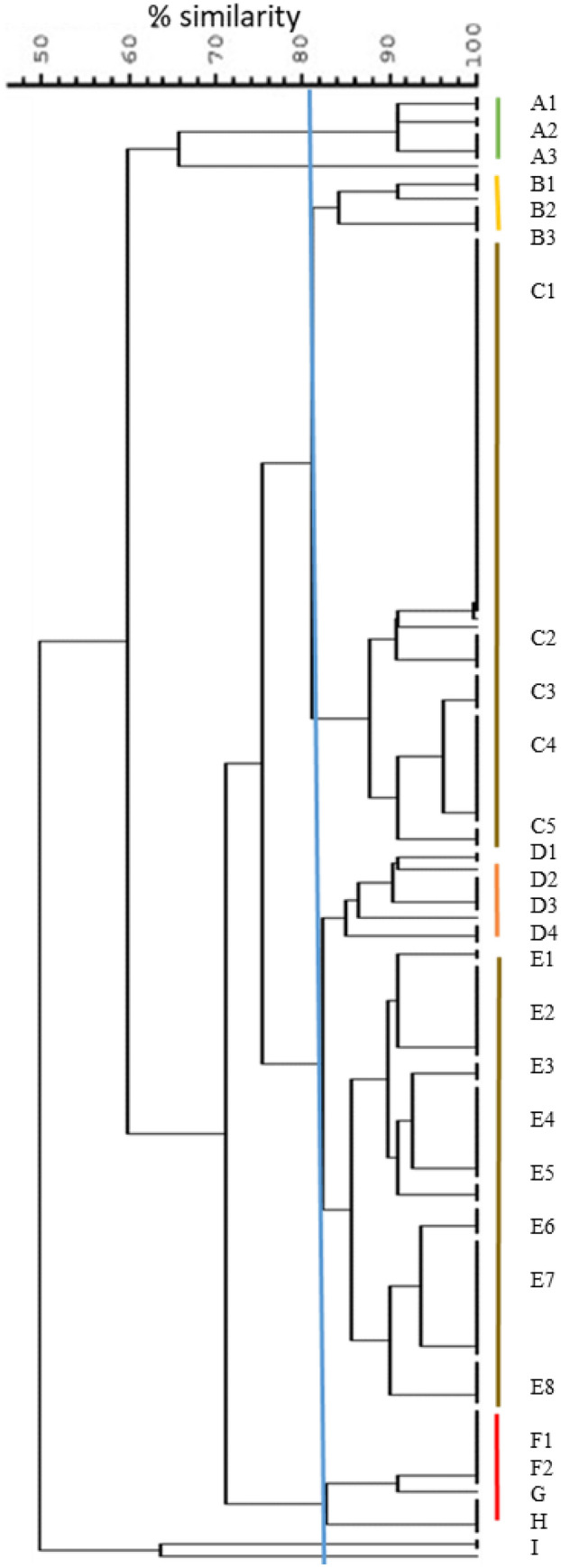
PFGE dendrogram. The tree was generated from composite fingerprinting with 80% similarity between the isolates. The pulsotypes are designated by the letters A through F, and individual patterns within these groups are numbered.

By PFGE, only 144 of the 166 isolates produced bands upon *Sma*I digestion (Supplementary Fig. S1). The number of fragments ranged from 8 to 13, with 31 distinct patterns. Nine pulsotypes were identified at an 80% similarity level (Fig. 4). The majority of the isolates, 71.6% (101/141), clustered into two major pulsotypes C and E, followed by A and D (14% of the isolates). Majority of the isolates from Gujarat (10/12, 83.3%), Karnataka (34/90, 37.7%) and Meghalaya (7/13, 53.8%) clustered into pulsotype C. For pulsotype E, the proportions were 100% (12/12) for Telangana, 26.6% (24/90) for Karnataka, 70% (7/10) for Uttar Pradesh and 46.1% (6/13) for Meghalaya isolates. All four isolates from Maharashtra belonged to pulsotype A (Table 1).

**Table 1:** State-wise details of isolates and their characteristics.

Sl. No	Isolate ID	Pulso type	OXA Disc	OXA MIC	*mec*A	SCC*mec* type	Toxin genes			spa type	ST	CC
							*hlg*	*tsst*	*pvl*			
1	GJ1	C1	S				+	−	−	NT	5407	8
2	GJ2	C1	S				−	−	−	t1965	5408	97
3	GJ4	D4	S				+	−	−	t021	5410	10
4	GJ5	C1	S				−	−	−	t1965	5408	97
5	GJ6	C1	S				+	−	−	t3092	72	8
6	GJ8	D4	S				−	−	−	t021	243	30
7	GJ9	C1	S				−	−	−	t3092	72	8
8	GJ10	C1	S				−	−	−	t1965	5411	97
9	GJ12	NT	S				−	−	−	t4522	2454	9
10	GJ13	C1	S				−	−	−	t4522	5411	97
11	GJ14	C1	S				−	−	−	t1965	5408	97
12	GJ17	C1	S				−	−	−	t3092	5413	8
13	GJ18	C1	S				−	−	−	t1965	5414	97
14	GJ22	NT	S				−	−	−	t1965	5413	97
15	GJ23	C1	S				−	−	−	t1965	4427	97
16	GJ24	NT	S				−	−	−	NT	5413	8
17	GJ25	C1	S				−	−	−	t1965	5414	97
18	GJ26	NT	S				−	−	−	NT	5413	97
19	GJ27	NT	S				−	−	−	NT	5414	97
20	KA1	C1	S				−	−	−	t359	2459	97
21	KA4	C1	S				+	−	+	t359	2459	97
22	KA6	C1	S				+	−	−	t359	2459	97
23	KA8	C1	S				+	−	−	t3992	2459	97
24	KA10	G	R	32	+	IVd	+	+	+	t3380	2459	97
25	KA12	C1	R	16			+	−	−	t4793	6	5
26	KA22	C1	S				+	−	−	t7287	2077	1
27	KA24	C2	R	16			+	−	−	t359	2459	97
28	KA25	C1	S				+	−	−	t359	2459	97
29	KA26	C1	R	16	+		+	−	+	t267	4992	97
30	KA31	C1	S				+	−	−	t359	2459	97
31	KA38	C1	S				+	−	−	t359	2459	97
32	KA41	F1	S				−	−	−	t7286	2454	9
33	KA42	C1	S				+	−	−	t359	2459	97
34	KA46	B2	S				+	−	+	t359	2459	97
35	KA48	C2	S	2	+	IVb	+	−	+	t359	2459	97
36	KA54	B2	R	16			+	−	+	NT	2459	97
37	KA55	NT	R	32	+		+	−	+	t359	2459	97
38	KA56	C1	S				+	−	+	t359	2458	97
39	KA57	C1	S				+	−	+	t359	2458	97
40	KA58	B2	S				−	−	+	t359	2459	97
41	KA59	C1	S				+	−	+	t359	2459	97
42	KA60	C1	R	32	+	IVb	−	−	+	t359	2459	97
43	KA62	C1	R	16	+		+	−	+	t359	2459	97
44	KA63	C2	S	1	+	IVb	+	−	+	t267	97	97
45	KA64	C1	S				+	−	+	t359	2459	97
46	KA66	C4	S				+	−	+	t359	2459	97
47	KA67	G	S				+	−	+	t267	1687	97
48	KA69	G	R	> 64	+	IVd	+	−	+	NT	NT	UA
49	KA70	C3	R	64			+	−	+	t267	1687	97
50	KA75	C4	S				+	−	+	t359	2459	97
51	KA76	G	S	1	+	V	+	−	+	NT	3881	1
52	KA77	C3	S	1	+	IVb	+	−	+	t2770	2459	97
53	KA78	C3	S	2	+	V	+	−	+	t267	1687	97
54	KA80	C3	S				+	−	+	t359	2459	97
55	KA81	NT	S	1	+	IVb	+	−	−	t359	2459	97
56	KA82	H	R	16	+		+	+	+	t359	2459	97
57	KA83	H	R	16	+		+	+	+	t359	2459	97
58	KA84	I	R	16	+	V	+	+	−	t359	2459	97
59	KA85	NT	S				+	−	+	t359	2459	97
60	KA86	C1	R	64	+	IVb	−	−	+	NT	NT	UA
61	KA90	NT	S	1	+	IVb	+	+	+	t7683	2459	97
62	KA94	E8	R	32	+	IVb	+	−	+	t008	6	5
63	KA103	F1	S				−	−	−	NT	2454	9
64	KA107	E8	R	16	+	IVb	−	−	−	t10760	4968	8
65	KA115	E7	S				+	+	−	UA	2454	9
66	KA119	F1	R	32	+		+	+	+	UA	672	UA
67	KA123	F1	S	1	+		−	−	−	t2297	2453	97
68	KA124	E7	S				+	+	+	t267	2453	97
69	KA128	F1	R	32	+		−	−	+	NT	NT	UA
70	KA131	E6	S				+	+	+	NT	2459	97
71	KA132	E6	S				+	+	+	t224	2459	97
72	KA133	B2	S	1	+		+	+	+	NT	1687	97
73	KA134	C5	S				+	+	+	t4522	2454	9
74	KA135	E2	R	16			+	+	+	t2700	2454	9
75	KA140	NT	S				+	+	+	t7867	2454	9
76	KA144	B1	S				+	+	+	t4522	2454	9
77	KA145	B1	S				+	+	+	NT	2454	9
78	KA148	E5	S				+	+	+	t16344	2454	9
79	KA149	NT	S				+	+	+	t7867	2454	9
80	KA150	E5	S				+	+	+	t16344	2454	9
81	KA151	NT	S				+	+	+	t3841	672	UA
82	KA152	E5	S				+	+	+	t3841	672	UA
83	KA154	NT	S	1	+	IVb	+	+	+	t021	1482	30
84	KA155	E7	S				+	+	+	t3841	672	UA
85	KA156	E7	S				+	+	+	NT	2454	9
86	KA159	E7	S				+	+	+	t359	2459	97
87	KA160	C1	S				+	−	+	t359	2459	97
88	KA161	C2	S				+	+	+	t359	2459	97
89	KA168	E3	S				+	−	+	t7696	1687	97
90	KA169	C3	R	64	+		−	−	+	t359	4975	9
91	KA170	C4	S				+	+	+	t10385	580	UA
92	KA171	F1	S				+	+	+	NT	2454	9
93	KA172	C4	S		+		+	−	+	t3841	672	UA
94	KA174	E3	S				+	+	+	t4522	2454	9
95	KA175	E7	R	16			+	+	+	t16344	2454	9
96	KA176	E7	R	32			+	+	+	t7867	9	1
97	KA177	F1	S				+	+	+	t2734	1687	97
98	KA180	E3	S				+	+	+	t4522	2454	9
99	KA181	E8	S				−	−	−	t519	4976	1
100	KA182	E7	S				+	+	+	t3841	672	UA
101	KA184	E7	S				+	+	+	NT	4976	1
102	KA192	D1	S				+	+	+	t18471	2454	9
103	KA193	E7	R	32	+		+	+	−	t4522	2454	9
104	KA194	A1	S				−	−	−	t359	5360	1
105	KA195	A3	S				+	−	−	t 668	5	5
106	KA196	D3	R	16			+	+	+	t002	3081	5
107	KA197	D3	S				−	−	−	t1236	97	97
108	KA198	E2	S				−	−	+	t1236	97	97
109	KA199	A1	S				−	−	+	t7287	5098	1
110	KA200	A1	R	32			+	−	+	t657	772	1
111	KA201	D4	R	16			+	−	+	t7287	5098	1
112	KA202	E2	S				+	−	+	t5019	2459	97
113	KA203	D1	S				+	+	+	t7867	2459	97
114	KA204	NT	S				−	−	−	t267	2459	97
115	KA205	NT	S				−	−	+	t18314	5418	9
116	KA206	E6	S				−	−	+	t3992	2459	97
117	KA207	D2	S				−	+	+	t3992	5689	97
118	MG2	C4	S				−	−	−	t7867	2454	9
119	MG3	C4	S	1	+	IVb	+	−	−	t7867	2454	9
120	MG4	NT	S				−	−	−	t4522	5416	9
121	MG5	C4	S	2	+	IVb	+	−	−	t17680	2454	9
122	MG6	C4	S				+	−	−	t7867	2454	9
123	MG7	C4	R	32	+	IVb	−	−	−	t7867	2454	9
124	MG8	C4	S	1	+	IVb	−	−	−	t1201	2459	97
125	MG9	NT	S	1	+	IVb	−	−	−	t1201	2454	9
126	MG10	E2	S	2	+	IVb	−	−	−	t4522	2454	9
127	MG11	C4	S				−	−	−	t17680	2454	9
128	MG15	E2	S				−	−	−	t4522	2454	9
129	MG16	E2	S				−	−	−	t7867	2454	9
130	MG19	E3	S	1	+	IVb	−	−	−	t2700	5418	9
131	MG25	E2	S				−	−	−	NT	5418	9
132	MG31	E7	S				−	−	−	t2700	NT	UA
133	MG32	NT	R	32			−	−	−	t657	5419	9
134	MG35	E2	S				−	−	−	t4522	2454	9
135	MG37	NT	S				−	−	−	t7867	2454	9
136	MG38	E2	S	1	+	IVb	−	−	−	t4522	2454	9
137	MH4	A3	S				+	+	−	t18320	2454	9
138	MH11	A3	S				−	−	−	t18320	2454	9
139	MH16	A2	S	1	+	IVb	+	+	−	t7867	2454	9
140	MH35	NT	S				+	+	+	NT	5113	9
141	MH39	A2	S				+	+	+	t7867	2454	9
142	TG2	E4	S				−	−	−	t7867	5419	9
143	TG3	E4	S				−	−	−	t7867	2454	9
144	TG4	E2	S	2	+	IVb	−	−	−	t4522	2454	9
145	TG5	E4	S				−	−	−	t17680	2454	9
146	TG6	E4	S				−	−	−	t7867	2454	9
147	TG7	E4	S				−	−	−	t7867	2454	9
148	TG8	E7	S				−	−	−	t1201	2459	97
149	TG9	E7	S				−	−	−	t1201	2219	97
150	TG10	E2	S	1	+	IVb	−	−	−	t4522	2454	9
151	TG11	E4	S				−	−	−	t17680	2454	9
152	TG13	E2	S				−	−	−	t4522	5420	9
153	TG14	E4	R	32	+	IVb	−	−	−	t7867	2454	9
154	TG15	E2	R	8	+	IVb	+	−	−	NT	2454	9
155	UP2	E1	S	1	+	IVb	−	−	−	t7867	2454	9
156	UP3	D3	R	> 64	+	IVb	−	−	−	t7867	2454	9
157	UP4	E1	S				−	−	−	t7867	2454	9
158	UP5	D3	S	1	+	IVb	−	−	−	t17680	2454	9
159	UP6	E4	S	2	+	IVb	−	+	−	t7867	2454	9
160	UP7	D3	R	64			−	−	−	t17680	2454	9
161	UP10	E4	S				−	−	−	t17680	2454	9
162	UP11	E4	S				−	−	−	NT	2454	9
163	UP13	NT	S				−	−	−	t4522	2454	9
164	UP14	E4	R	64			−	−	−	t7867	2454	9
165	UP15	NT	R	64			−	−	−	t4522	2454	9
166	UP16	E4	S				−	−	−	t7867	2454	9

CC = Clonal complex; GJ = Gujarat; KA = Karnataka; MG = Meghalaya; MH = Maharashtra; MIC = minimum inhibitory concentration; OXA = oxacillin; NT = Non-typable; ST = Sequence Type; TG = Telangana; UA = Unassigned; UP = Uttar Pradesh.

To examine virulence determinants with the potential for pathogenicity, we subjected the isolates to disk diffusion test, broth microdilution for MIC for determining susceptibility to oxacillin and to PCR for *mec*A and *mec*C genes. Out of the 166 isolates, 34 (20.4%) showed resistance to oxacillin, with MICs of 8 to 64 µg/mL, whereas *mec*A amplification was observed with 44 (26.5%) isolates (Supplementary Table S3); none of the isolates contained *mec*C (data not shown). Out of the 44 *mec*A-positive isolates, 20 showed oxacillin MIC of > 4 µg/mL and 14 isolates showed oxacillin resistance in the absence of *mec*A or *mec*C (Table 1). By the definition of oxacillin susceptibility and presence of *mec*A, 24 of the 166 isolates (14.46%) could be categorised as OS-MRSA (Supplementary Table S4). Among 44 *mec*A^+^ isolates, 32 were typeable for SCC*mec* I to V, and most of the isolates (26/32) belonged to SCC*mec* IVb, whereas 12 isolates did not amplify any bands for these types (Table 1). The predominant MRSA clones were CC9-ST2454-SCC*mec*IVb-t7867 and CC97-ST2459-SCC*mec*IVb-t359 (Tables 1, 2).

**Table 2:** Prevalence of different clones among MRSA, OS-MRSA and MSSA.

State	MRSA		OS-MRSA		MSSA	
	Prevalent clones	Major pulsotype	Prevalent clones	Major pulsotype	Prevalent clones	Major pulsotype
Gujarat	None	None	None	None	CC97/diverse-STs/t1965	C
					CC8/diverse-STs/t30921	None
Karnataka	CC97/2459/t359	C & H	None	None	CC97/2459/t359	C
	CC9/2454/diverse-spa-types	E	CC97/2459-IVb/diverse-spa-types	C	CC9/2454/t4522	E
	CC1/diverse-ST/diverse-spa-types	Diverse			CC1/diverse-STs/diverse-spa-types	A & E
	CC5/6/diverse-spa-types					
Meghalaya	CC9/2454/t7867	C	CC9/2454-IVb/diverse-spa-types	C	CC9/2454/t7867 or t4522	C & E
Maharashtra	None	None	CC9/2454-IVb/t7867	A	CC9/2454/t18320	A
Telangana	CC9/2454-IVb/t7867	E	CC9/2454-IVb/t4522	E	CC9/2454/t7867	E
Uttar Pradesh	CC9/2454/t7867	D	CC9/2454-IVb/t7867	E	CC9/2454/t7867	E

PCR was also carried out to test the presence of the toxin genes *hlg*, *tsst* and *pvl*, which encode for the most common virulence determinants. The results showed that 56.25%, 28.75% and 47.5% of the isolates harboured *hlg*, *tsst* and *pvl* genes, respectively (Table 1). Notably, a high proportion of Karnataka isolates harboured *hlg* (87.7%) and *pvl* (82.2%) genes. Among these, isolates belonging to pulsotypes C2 and C3 had both *hlg* and *pvl* genes, and pulsotypes E5, E6 and E7 were positive for all the three genes (Table 1)*.* All of the B, G, H and I pulsotypes carried the *pvl* gene, although there were only one to six isolates in these pulsotypes, and among pulsotypes C and E, which each contained more than 50 isolates, *pvl* carriage ranged between 39.62% and 44.23% (Table 1).

## Discussion

Mastitis is a major concern for the dairy industry, as it not only affects animal’s health, but also results in direct and indirect losses^33^. Despite producing highest amount of milk, Indian dairy industry is crippled by mastitis^34^, with an estimated annual loss of INR 71.6551 billion (USD 1.6 billion) as of two decades ago^35^. Loss per incidence of INR 400 to 2086 (about USD 6 to 31) and annual loss per animal of INR 1500 to 1700 (about USD 22 to 25) have been reported^36–39^.

Although bovine mastitis is a multi-factorial, multi-etiological disease, *S. aureus* is one of the most common pathogens associated with it worldwide, including India^40–42^. Infection with *S. aureus* can lead to a wide range of manifestations including clinical as well as subclinical, chronic, persistent and recurrent mastitis. Transmission of *S. aureus* usually occurs from one animal to another through milking, but can also occur through contagion. Complete information on the population structure, virulence and resistance characteristics of mastitis-associated *S. aureus* is desired to monitor its dissemination among animal populations and for risk assessment, so as to improve interventions. This is the first study to report the genotypes of bovine mastitis-associated *S. aureus* from different locations in India, through the simultaneous application of *spa* typing, MLST and PFGE.

Among several genotypes of *S. aureus,* only a few appear to be associated with bovine mastitis^43^. As far as *spa* types are concerned, from bovine milk, t002, t008, t267, t359 and t6877 have been reported from Brazil, Canada and Japan^44–47^; t127 and t2279 were reported to be common in China^48^. The *spa* types t267 and t359 were also observed to be predominant among our isolates from Karnataka, and have been previously reported from this state^29^; other types such as t7867 and t3841 are also common in Karnataka^30^. On the other hand, t7867 was observed in all the states, suggesting that t267, t359 and t7867 may be commonly associated with bovine mastitis in India.

We observed a predominance of ST2454 and ST2459 among our isolates, although there were also 12 newly identified STs. Other types, ST72, ST245, ST239 and ST1687 have been previously reported from Andhra Pradesh, Telangana and Karnataka^29^, implying the existence of diverse genetic populations. Integrated data of our isolates showed that CC97-ST5414-t1965 and CC97-ST2459-t359 were predominant in the states of Gujarat and Karnataka, respectively, whereas CC9-ST2454-t7867 was frequent among isolates of the remaining four states studied.

Early phenotypic studies from Europe and the US showed low prevalence of MRSA among livestock^13–15,18^. By specific culturing or PCR for *mec*A or *mec*C, other studies have reported a relatively lower prevalence of 0% to 9.7% from Europe, US, Japan and South Korea^17,19,20,25,26,45^, while higher prevalence ranging from 16.7 to 49.6% has been reported from Germany, Brazil, Japan, Sweden and China^16,21–24^. We observed that 12.04% of our 166 isolates were methicillin-resistant both phenotypically and genotypically, whereas 14.46% were OS-MRSA. In total, 58 isolates (34.94%) were either oxacillin-resistant or *mec*A^+^. Earlier studies from India, which have performed either phenotypic or genotypic tests (not both), have reported a range from 5 to 27%^49–52^. It is possible that the high levels (34.94%) of MRSA within our isolates could just be a consequence of the application of both genopytic and phenotypic tests, but is similar to higher proportions observed in several parts of the world as stated above. It may, however, be noted that defining methicillin resistance has been problematic since a variety of conditions such as modifications in native penicillin-binding proteins, hyper-production of β-lactamase, or production of methicillinase can lead to resistance^53^. These studies together suggest that classifying an isolate as MRSA is complex, and in the context of a clinical setting, the phenotypic assay may be more important, although it is possible that genotypically positive, phenotypically negative isolates may still gain resistance in response to environmental stimuli such as encounter with salts or antibiotics^54–56^.

A large majority of our isolates, irrespective of methicillin resistance/susceptibility, clustered among CC97 and CC9. Historically, livestock-associated MRSA (LA-MRSA) were first reported from pig production systems, and found to belong to ST398^57^. Later, ST398 was also reported from bovine mastitis cases^58,59^, as well as other samples from various other livestock^57^. Majority of bovine mastitis-associated MRSA reported worldwide belong to CC398^60–63^, whilst less represented clones include those belonging to CC1, CC5, CC9, CC97, CC130 and a few others^64–67^. It is now apparent that strains belonging to CC9 are the predominant LA-MRSA in Asian countries whereas ST398 is predominant in Europe and North America^2,60–63^. In addition, CC97 MRSA have also been demonstrated to be predominant in dairy cattle and pigs in Italy^68^. On the other hand, a large proportion of MSSA from a number of countries have been reported to belong to CC398 and CC97^68–77^. ST398, an ST which clusters under CC398, is also predominant among MSSA from Japan and China^78,79^. In addition, MSSA from China also belonged to ST97 and ST9-SCC*mec*XII-t899 whilst MRSA belonged to ST1-SCC*mec*I-t1939^62,80^. Whereas CC97 and CC9 were widely represented in our MRSA as well as MSSA isolates, we did not find any isolate belonging to ST398, suggesting commonality as well as variance with studies worldwide.

The exact origin of the predominant strains in our study is not discernible since neither this is a longitudinal study, nor is there sufficient data from within India to compare and analyse. One of the major cluster of strains from our study was CC9-ST2454. This particular clone has been previously reported from pigs in China and from water buffalos in the Philippines^81,82^. It may be argued that our CC9-ST2454 strains originated from pigs. However, most of the places from where isolates were obtained for our study were from semi-urban or village areas from states which have very less pig population. In India, the majority of pig rearing occurs in the Northeast region and the state of Kerala. In fact, a little more than 70% of the pig population of India is in the Northeast region, and only the state of Meghalaya belonged to this geographical location within the isolates that we obtained. In addition, most of the other locations from where isolates were obtained were free from domesticated or feral pigs. Hence, we consider it highly unlikely that ST2454-SCC*mec*IVb MRSA in our study may have originated directly from pigs, although we cannot rule out transmission events which predate isolation of the organisms used in our study.

Majority (26/32) of our *mec*A-containing isolates were typed as SCC*mec*IV, which has also been reported in other studies elsewhere^83,84^. On the other hand, several recent studies have reported on oxacillin-susceptible *mec*A^+^ *S. aureus* (OS-MRSA)^22,85–89^. In our study, 14.46% of the isolates were OS-MRSA. In Karnataka, OS-MRSA belonged to CC97-ST2459-SCC*mec*IVb with diverse *spa* types. In other states, OS-MRSA belonged to CC9-ST2454-SCC*mec*IVb-t7867 or t4522. A report from China identified high incidence (35.9%) of OS-MRSA, and with SCC*mec*V-t267 whereas OS-MRSA isolates from Shanghai belonged to different *spa* and SCC*mec* types^22^. One study from India reported 48.7% OS-MRSA, with diverse STs and *spa* types^87^. Only two of our OS-MRSA isolates belonged t267 and the remaining belonged to diverse types indicating clonal diversity among OS-MRSA.

By PFGE, we observed 31 pulsotypes among 144 isolates. Comparatively, narrower as well as wider ranges (from 10 among 146 isolates to 33 among 66 strains to 39 among 245 strains^10,90^) have been reported by others. Although PFGE has been projected to be the gold standard for non-sequence-based typing of bacterial strains, there are very few PFGE typing studies for bovine mastitis-associated *S. aureus* isolates, possibly owing to the cost of the equipment and the requirement for technical expertise. In addition, the results of PFGE typing for strains from different laboratories is not easily comparable without an extensive cataloguing of the banding patterns. It may, however, be noted that the majority of ST2454 and ST2459 strains clustered to pulsotypes E and C (30/57 and 21/40), respectively, suggesting corroboration between the two typing methods.

A large proportion of the isolates from Karnataka showed the presence of both *hlg* and *pvl* genes, suggesting pathogenic potential of these isolates. It has been hypothesized that different clones might display differential patterns of antimicrobial resistance and virulence factors^48^. We observed that isolates expressing the toxin genes *hlg*, *tsst* and *pvl* belonged to particular pulsotypes (C2, C3, E5, E6 and E7), lending credence to this hypothesis. It should be noted that C and E pulsotypes encompassed the largest groups with 52 and 53 strains, respectively, but only five sub-pulsotypes among these contained the majority of the isolates carrying the *pvl* gene, suggesting potential association between certain pulsotypes and virulence.

In conclusion, the majority of the isolates that we studied belonged to two major clonal complexes CC97 and CC9, similar to what has been observed globally. And yet, genetic diversity was evident as supported by the existence of more than 30 different genetic types (by any of the three typing methods). While heterogeneity at the micro-level would appear to support our hypothesis that mastitis-associated *S. aureus* strains from India show diversity, the diversity appeared to be within the two CCs. Among the oxacillin-resistant strains, the majority of the isolates belonged to CC97-ST2454-SCC*mec*IVb-t359 in Karnataka and CC9-ST2454-SCC*mec*IVb-t7867 in the other states except Maharashtra. These strains may therefore represent the predominant clones in these regions. Importantly, ST2454- and ST2459-SCC*mec*IVb appear to be the major MRSA clones in the country. However, a wider inference of the study is limited by the higher number of isolates from some states than others. Hence, more extensive, structured studies involving more number of states and isolates are required to better understand the genetic diversity of *S. aureus* isolates causing mastitis in India. This could help in elucidating strain variation, studying movement and transmission events, and in designing better control and prevention strategies for bovine mastitis.

## Materials and methods

### Isolates and genomic DNA isolation

For this study, 166 isolates of *S. aureus* curated at the Department of Microbiology, Veterinary College, Bangalore, Department of Microbiology, Veterinary College, Shivamogga, Karnataka State, and the National Institute of Animal Biotechnology, Hyderabad, were used. The study was therefore retrospective, and focussed on obtaining complete set of typing data for as many isolates as possible. The isolates had been obtained at various times from clinical and subclinical mastitis cases from six different states: Gujarat (Anand), Karnataka (North and South), Maharashtra (Mumbai), Meghalaya (unknown locations), Telangana (Hyderabad) and Uttar Pradesh (unknown locations) in India. Whereas details of the locations and dates are available for most of the isolates from Karnataka (Supplementary Table S1), no details are available for other locations as the isolates were obtained from collaborators. The isolates had been identified earlier as *S. aureus* by biochemical tests as well as by polymerase chain reaction (PCR), and were re-confirmed before beginning this study by PCR for the *nuc* gene^91^.

The isolates were cultured for 24 h at 35 °C on tryptic soy agar plates, and a single colony was further propagated in tryptic soy broth for 24 h. The cells were pelleted and genomic DNA was extracted using the HiYield Genomic DNA Mini Kit (Bacteria) (Real Biotech Corporation, Taiwan).

### Spa typing

The *spa* repeat region was amplified using primers and conditions as per RidomSpa server [spa-1113f. (5′-TAAAGACGATCCTTCGGTGAGC-3′) and spa-1514r (5′-CAGCAGTAGTGCCGTTTGCTT-3′)]^92^. PCR was performed with genomic DNA (500 ng), dNTPs (200 µM), each primer (10 pmol), and *Taq* DNA polymerase (1.25 U; New England Biolabs). Thermal cycling included initial denaturation (5 min at 80 °C) followed by 35 cycles of denaturation (45 s at 94 °C), annealing (45 s at 60 °C), and extension (90 s at 72 °C), with a single final extension (10 min at 72 °C). A portion of the PCR product was run on 1.5% agarose gel, and the remaining was purified using a kit (Real Biotech Corporation) and sequenced (Bioserve Biotechnologies, Hyderabad, India). Both forward and reverse reads were analyzed using Ridom SpaServer (https://spa.ridom.de/index.shtml) and spaTyper (http://spatyper.fortinbras.us/).

### Multi-locus sequence typing and analysis

The designated seven housekeeping genes of *S. aureus* were amplified by PCR with the specific primers as prescribed by Enright et al.^93^ (Staph MLST database). PCRs was performed with genomic DNA (1 μg of), primers (500 ng each), and Master Mix (Takara Bio). The thermal cycling included an initial denaturation at 95 °C for 5 min, then 30 cycles of annealing at 55 °C for 1 min, extension at 72 °C for 1 min, and denaturation at 95 °C for 1 min, followed by a final extension of 72 °C for 5 min. The amplified fragments were purified and sequenced. Consensus sequences were obtained by aligning sequences from both orientations and analyzed using the PubMLST database to determine the ST. Minimum spanning tree was constructed based on STs and the advanced cluster analysis was performed to define the clonal complexes (CCs) by using the BioNumerics software version 7 (Applied Maths NB, Belgium). A CC was defined by similarity in at least four or more loci.

### Pulsed-field gel electrophoresis

The bacterial isolates were grown for 24 h at 37 °C on tryptic soy agar plates. 1.8% SeaKem gold agarose was boiled in TE buffer (10 mM Tris–HCl, pH 8.0, 1 mM EDTA), aliquoted into micro-centrifuge tubes, and equilibrated to 60 °C in a heat block. Three to five colonies of each bacterial isolate were suspended in TEN buffer (0.1 M Tris–HCl, 0.15 M NaCl, 0.1 M EDTA; 125 µL) and adjusted to approx. 3.0 McFarland. Lysostaphin (5 µL of 1 mg/Ml; Sigma-Aldrich, India) was added to the cell suspension followed by the addition of 1.8% SeaKem gold agarose (125 µL) and transferred to the plug mould. Solidified plugs were placed in EC buffer (6 mM Tris–HCl, 1.0 M NaCl, 0.1 M EDTA, 0.5% Brij 58, 0.2% deoxycholate, 0.5% sarkosyl; 1 mL) and incubated in a 37 °C water bath for 4 h. The EC buffer was replaced with ESP buffer (10 mM Tris–HCl, 1.0 mM EDTA, 1% SDS, 1 mg/mL of proteinase K, pH 8.0; 1 mL) and incubated overnight at 55 °C in a water bath. The ESP buffer was removed and the plugs were washed three times for 30 min each in TE buffer (10 mL). Plugs were digested with *Sma*I (30 U; New England Biolabs) for 2 to 3 h at room temperature, and then sealed with 1% of Seakem GTG agarose in 0.5X TBE buffer (0.045 M Tris base, 0.045 M boric acid, pH 8.3, 1 mM EDTA). Electrophoresis was performed in CHEF-DRIII PFGE system (Bio-Rad Laboratories) with 5 s initial switch time and 40 s final switch time for 20 h at 120° angle, with 6-V/cm gradient, and chiller set at 14 °C. After electrophoresis, the gel was stained with ethidium bromide solution (1 mg/mL) and de-stained in water. The gel image was captured and subjected to analysis using BioNumerics software version 7. The percent similarity was attained on DICE coefficients derived from the unweighted pair group method with arithmetic averages (UPGMA). A coefficient similarity of 80% was fixed to assemble PFGE clusters. Band position tolerance was set at 1.0%.

### Evaluation of methicillin susceptibility and the presence of *mec* genes

For these studies, methicillin-sensitive ATCC-29213 and methicillin-resistant ATCC-335913 strains of *S. aureus* were used as control strains. The antibiotic susceptibility of the isolates was evaluated by the disk diffusion test according to the Kirby-Bauer method^94^. Each isolate was subjected to testing three times along with a methicillin-sensitive and a –resistant reference strain (ATCC 29,213 and 335,913, respectively). Briefly, the isolates were inoculated into brain heart infusion broth (1 Ml; HiMedia) and incubated overnight at 37 °C. A suspension (0.5 mL) of the resultant culture was adjusted to 0.5 on McFarland scale using sterile phosphate-buffered saline (PBS, pH 7.2), and then spread plated onto Mueller–Hinton agar (HiMedia). Discs containing oxacillin or cefoxitin (HiMedia), as well as a sterile disc serving as a negative control, were gently placed on the dried inoculum, and the plates were incubated at 37 °C for 24 h. The zone of inhibition, inclusive of borders, was measured in mm and the breakpoints were applied as recommended for veterinary pathogens by the Clinical and Laboratory Science Institute^95^.

All the isolates were subjected to amplification of *mec*A and *mec*C genes using primers and conditions described previously^96^. All the isolates which showed *mec*A amplification were subjected to SCC*mec* typing using multiplex PCR for SCC*mec* types I to V as described previously^97^.

Further, minimum inhibitory concentration (MIC) of oxacillin was determined for all the isolates which showed either *mec*A amplification or resistance in disc diffusion, following the broth micro-dilution method recommended by CLSI^95^.

### Detection of toxin genes

Presence of toxin genes (*hlg, tsst* and *pvl*) was confirmed by individual PCRs using DNA template (100 ng) and gene-specific primer pairs (0.5 μM of each) added to a PCR Master Mix (Takara Bio, Japan), and amplification was performed with the following cycle: 15 min at 94 °C, followed by 30 cycles of 30 s at 94 °C, 1 min at 53 °C, and 1 min at 72 °C, with a final 10 min elongation step at 72 °C. PCR products were analysed by 2% agarose gel electrophoresis and UV transillumination. The primers were F: GCCAATCCGTTATTAGAAAATGC and R: CCATAGACGTAGCCACGGAT for *hlg*, F: ACCCCTGTTCCCTTATCATC and R: TTTTCAGTATTTGTAACGCC for *tsst*, and F: GGCGCTGAGGTAGTCAAAAG and R: TCGGAATCTGATGTTGCAGT for *pvl*^98,99^.

## Supplementary Information

Supplementary Information.

## Data Availability

All data generated or analysed during this study are included in this article and its Supplementary Information files.
