# Supplementary material for: Molecular fingerprinting of bovine mastitis-associated Staphylococcus aureus isolates from India
Source: Sci Rep. 2021 Jul 27;11:15228. doi: 10.1038/s41598-021-94760-x (PMC8316343; doi:10.1038/s41598-021-94760-x)
Supplement: Supplementary file 1 — Supplementary Information. [file 41598_2021_94760_MOESM1_ESM.pdf]

**Supplementary Table 1. Details for locations in Karnataka from where milk samples were collected to obtain the isolates.**

| Isolate ID | Date of collection | Farm type | Location | Taluk | District | Region of the State |
|---|---|---|---|---|---|---|
| KA1 | 17-09-2009 | Organized | Huskur | Doddaballapura | Bengaluru Rural | Southeast |
| KA4 | 17-09-2009 | Organized | Huskur | Doddaballapura | Bengaluru Rural | Southeast |
| KA6 | 06-10-2009 | Organized | Bidar | Bidar | Bidar | Northeast |
| KA8 | 05-11-2009 | Unorganized | Chikkanayakanahalli | Chikkanayakanahalli | Tumkur | Southeast |
| KA10 | 05-11-2009 | Unorganized | Chikkanayakanahalli | Chikkanayakanahalli | Tumkur | Southeast |
| KA12 | 05-11-2009 | Unorganized | Chikkanayakanahalli | Chikkanayakanahalli | Tumkur | Southeast |
| KA22 | 05-11-2009 | Unorganized | Chikkanayakanahalli | Chikkanayakanahalli | Tumkur | Southeast |
| KA24 | 05-11-2009 | Unorganized | Chikkanayakanahalli | Chikkanayakanahalli | Tumkur | Southeast |
| KA25 | 05-11-2009 | Unorganized | Chikkanayakanahalli | Chikkanayakanahalli | Tumkur | Southeast |
| KA26 | 05-11-2009 | Unorganized | Chikkanayakanahalli | Chikkanayakanahalli | Tumkur | Southeast |
| KA31 | 05-11-2009 | Unorganized | Chikkanayakanahalli | Chikkanayakanahalli | Tumkur | Southeast |
| KA38 | 06-10-2009 | Organized | Bidar | Bidar | Bidar | Northeast |
| KA41 | 15-12-2009 | Organized | Hessaraghatta | Bengaluru North | Bengaluru Urban | Southeast |
| KA42 | 15-12-2009 | Organized | Hessaraghatta | Bengaluru North | Bengaluru Urban | Southeast |
| KA46 | 15-12-2009 | Organized | Hessaraghatta | Bengaluru North | Bengaluru Urban | Southeast |
| KA48 | 15-12-2009 | Organized | Hessaraghatta | Bengaluru North | Bengaluru Urban | Southeast |
| KA55 | 20-10-2010 | Unorganized | Ballur | Anekal | Bengaluru Rural | Southeast |
| KA56 | 20-10-2010 | Unorganized | Ballur | Anekal | Bengaluru Rural | Southeast |
| KA57 | 20-10-2010 | Unorganized | Ballur | Anekal | Bengaluru Rural | Southeast |
| KA58 | 20-10-2010 | Unorganized | Ballur | Anekal | Bengaluru Rural | Southeast |
| KA59 | 20-10-2010 | Unorganized | Ballur | Anekal | Bengaluru Rural | Southeast |
| KA60 | 20-10-2010 | Unorganized | Ballur | Anekal | Bengaluru Rural | Southeast |
| KA61 | 20-10-2010 | Unorganized | Ballur | Anekal | Bengaluru Rural | Southeast |
| KA62 | 20-10-2010 | Unorganized | Ballur | Anekal | Bengaluru Rural | Southeast |
| KA63 | 20-10-2010 | Unorganized | Ballur | Anekal | Bengaluru Rural | Southeast |

| | | | | | | |
|---|---|---|---|---|---|---|
| KA64 | 20-10-2010 | Unorganized | Ballur | Anekal | Bengaluru Rural | Southeast |
| KA66 | 20-10-2010 | Unorganized | Ballur | Anekal | Bengaluru Rural | Southeast |
| KA67 | 20-10-2010 | Unorganized | Ballur | Anekal | Bengaluru Rural | Southeast |
| KA69 | 20-10-2010 | Unorganized | Ballur | Anekal | Bengaluru Rural | Southeast |
| KA70 | 20-10-2010 | Unorganized | Ballur | Anekal | Bengaluru Rural | Southeast |
| KA75 | 20-10-2010 | Unorganized | Thammanayakanalli | Anekal | Bengaluru Rural | Southeast |
| KA76 | 20-10-2010 | Unorganized | Thammanayakanalli | Anekal | Bengaluru Rural | Southeast |
| KA77 | 20-10-2010 | Unorganized | Thammanayakanalli | Anekal | Bengaluru Rural | Southeast |
| KA78 | 20-10-2010 | Unorganized | Thammanayakanalli | Anekal | Bengaluru Rural | Southeast |
| KA80 | 20-10-2010 | Unorganized | Thammanayakanalli | Anekal | Bengaluru Rural | Southeast |
| KA81 | 20-10-2010 | Unorganized | Thammanayakanalli | Anekal | Bengaluru Rural | Southeast |
| KA82 | 20-10-2010 | Unorganized | Thammanayakanalli | Anekal | Bengaluru Rural | Southeast |
| KA83 | 20-10-2010 | Unorganized | Thammanayakanalli | Anekal | Bengaluru Rural | Southeast |
| KA84 | 20-10-2010 | Unorganized | Thammanayakanalli | Anekal | Bengaluru Rural | Southeast |
| KA85 | 20-10-2010 | Unorganized | Thammanayakanalli | Anekal | Bengaluru Rural | Southeast |
| KA86 | 20-10-2010 | Unorganized | Thammanayakanalli | Anekal | Bengaluru Rural | Southeast |
| KA90 | 20-10-2010 | Unorganized | Thammanayakanalli | Anekal | Bengaluru Rural | Southeast |
| KA94 | 20-10-2010 | Organized | Ballur | Anekal | Bengaluru Rural | Southeast |
| KA103 | 24-01-2011 | Organized | Hameelpura | Bidar | Bidar | Northeast |
| KA107 | 24-01-2011 | Organized | Hameelpura | Bidar | Bidar | Northeast |
| KA115 | 25-01-2011 | Organized | Bidar | Bidar | Bidar | Northeast |
| KA119 | 25-01-2011 | Organized | Bidar | Bidar | Bidar | Northeast |
| KA123 | 26-01-2011 | Organized | Bidar | Bidar | Bidar | Northeast |
| KA124 | 26-01-2011 | Organized | Bidar | Bidar | Bidar | Northeast |
| KA128 | 02-02-2011 | Unorganized | Gunnahalli | Bidar | Bidar | Northeast |
| KA131 | 02-02-2011 | Unorganized | Gunnahalli | Bidar | Bidar | Northeast |
| KA132 | 02-02-2011 | Unorganized | Gunnahalli | Bidar | Bidar | Northeast |
| KA133 | 02-02-2011 | Unorganized | Gunnahalli | Bidar | Bidar | Northeast |
| KA134 | 02-02-2011 | Unorganized | Gunnahalli | Bidar | Bidar | Northeast |

| | | | | | | |
|---|---|---|---|---|---|---|
| KA135 | 02-02-2011 | Unorganized | Gunnahalli | Bidar | Bidar | Northeast |
| KA138 | 02-02-2011 | Unorganized | Gunnahalli | Bidar | Bidar | Northeast |
| KA140 | 02-02-2011 | Unorganized | Gunnahalli | Bidar | Bidar | Northeast |
| KA144 | 02-02-2011 | Unorganized | Gunnahalli | Bidar | Bidar | Northeast |
| KA145 | 02-02-2011 | Unorganized | Gunnahalli | Bidar | Bidar | Northeast |
| KA148 | 04-02-2011 | Unorganized | Mandakanalli | Bidar | Bidar | Northeast |
| KA149 | 04-02-2011 | Unorganized | Mandakanalli | Bidar | Bidar | Northeast |
| KA150 | 04-02-2011 | Unorganized | Mandakanalli | Bidar | Bidar | Northeast |
| KA151 | 04-02-2011 | Unorganized | Mandakanalli | Bidar | Bidar | Northeast |
| KA152 | 04-02-2011 | Unorganized | Mandakanalli | Bidar | Bidar | Northeast |
| KA154 | 04-02-2011 | Unorganized | Mandakanalli | Bidar | Bidar | Northeast |
| KA155 | 04-02-2011 | Unorganized | Mandakanalli | Bidar | Bidar | Northeast |
| KA156 | 04-02-2011 | Unorganized | Mandakanalli | Bidar | Bidar | Northeast |
| KA159 | 02-02-2011 | Unorganized | Jajanmugali | Basavakalyana | Bidar | Northeast |
| KA160 | 02-02-2011 | Unorganized | Jajanmugali | Basavakalyana | Bidar | Northeast |
| KA161 | 02-02-2011 | Unorganized | Jajanmugali | Basavakalyana | Bidar | Northeast |
| KA168 | 04-02-2011 | Unorganized | Kadwad | Bidar | Bidar | Northeast |
| KA169 | 04-02-2011 | Unorganized | Kadwad | Bidar | Bidar | Northeast |
| KA170 | 04-02-2011 | Unorganized | Kadwad | Bidar | Bidar | Northeast |
| KA171 | 04-02-2011 | Unorganized | Kadwad | Bidar | Bidar | Northeast |
| KA172 | 04-02-2011 | Unorganized | Kadwad | Bidar | Bidar | Northeast |
| KA174 | 04-02-2011 | Unorganized | Kadwad | Bidar | Bidar | Northeast |
| KA175 | 04-02-2011 | Unorganized | Kadwad | Bidar | Bidar | Northeast |
| KA176 | 04-02-2011 | Unorganized | Kadwad | Bidar | Bidar | Northeast |
| KA177 | 04-02-2011 | Unorganized | Kadwad | Bidar | Bidar | Northeast |
| KA180 | Unknown | Clinic | Bidar | Bidar | Bidar | Northeast |
| KA181 | Unknown | Clinic | Bidar | Bidar | Bidar | Northeast |
| KA182 | Unknown | Clinic | Bidar | Bidar | Bidar | Northeast |
| KA184 | Unknown | Clinic | Bidar | Bidar | Bidar | Northeast |

| | | | | | | |
|---|---|---|---|---|---|---|
| KA192 | Unknown | Clinic | Bidar | Bidar | Bidar | Northeast |
| KA193 | Unknown | Clinic | Bidar | Bidar | Bidar | Northeast |
| KA194 | Unknown | Clinic | Bidar | Bidar | Bidar | Northeast |
| KA195 | Unknown | Unorganized | Uppinangadi | Puttur | Dakshina Kannada | Southwest |
| KA196 | Unknown | Unorganized | Uppinangadi | Puttur | Dakshina Kannada | Southwest |
| KA197 | Unknown | Unorganized | Uppinangadi | Puttur | Dakshina Kannada | Southwest |
| KA198 | Unknown | Unorganized | Uppinangadi | Puttur | Dakshina Kannada | Southwest |
| KA199 | Unknown | Unorganized | Uppinangadi | Puttur | Dakshina Kannada | Southwest |
| KA200 | Unknown | Unorganized | Uppinangadi | Puttur | Dakshina Kannada | Southwest |
| KA201 | Unknown | Unorganized | Uppinangadi | Puttur | Dakshina Kannada | Southwest |
| KA202 | Unknown | Unorganized | Shivamogga | Shivamogga | Shivamogga | Central |
| KA203 | Unknown | Unorganized | Shivamogga | Shivamogga | Shivamogga | Central |
| KA204 | Unknown | Unorganized | Shivamogga | Shivamogga | Shivamogga | Central |
| KA205 | Unknown | Unorganized | Shivamogga | Shivamogga | Shivamogga | Central |
| KA206 | Unknown | Unorganized | Shivamogga | Shivamogga | Shivamogga | Central |
| KA207 | Unknown | Unorganized | Shivamogga | Shivamogga | Shivamogga | Central |

Notes:

1. The locations in the Northeast and the Southeast are separated by at least 600 km. The Southwest and Central locations are each about 300 km from the locations in the Southeast. The Southwest and Central locations are about 800 and 650 km, respectively, from the Northeast location.
2. Unorganized means small holding of a few animals by farmers.

**Supplementary Table S2**. **Newly identified ST profiles in the study**

| Isolate No. | ST | CC | arcC | aroE | glpF | gmk | pta | tpi | yqiL |
|---|---|---|---|---|---|---|---|---|---|
| GJ1 | 5407 | 8 | 3 | 3 | 1 | 1 | 8 | 8 | 6 |
| GJ2 | 5408 | 97 | 3 | 1 | 487 | 1 | 4 | 5 | 3 |
| GJ4 | 5410 | 30 | 2 | 2 | 5 | 2 | 4 | 3 | 3 |
| GJ5 | 5408 | 97 | 3 | 1 | 487 | 1 | 1 | 5 | 3 |
| GJ10 | 5411 | 97 | 3 | 1 | 487 | 1 | 1 | 5 | 10 |
| GJ13 | 5411 | 97 | 3 | 1 | 487 | 1 | 1 | 5 | 10 |
| GJ14 | 5408 | 97 | 3 | 1 | 487 | 1 | 1 | 5 | 3 |
| GJ17 | 5413 | 8 | 1 | 4 | 1 | 8 | 4 | 4 | 9 |
| GJ18 | 5414 | 97 | 3 | 1 | 487 | 1 | 1 | 5 | 96 |
| GJ25 | 5408 | 97 | 3 | 1 | 487 | 1 | 1 | 5 | 3 |
| GJ26 | 5415 | 97 | 1 | 1 | 487 | 8 | 1 | 5 | 3 |
| MG19 | 5418 | 9 | 3 | 3 | 1 | 1 | 1 | 4 | 10 |
| MG25 | 5418 | 9 | 3 | 3 | 1 | 1 | 1 | 4 | 10 |
| MG32 | 5419 | 9 | 3 | 3 | 645 | 1 | 264 | 1 | 13 |
| TG2 | 5419 | 9 | 3 | 3 | 645 | 1 | 264 | 1 | 13 |
| TG13 | 5420 | 9 | 3 | 3 | 487 | 1 | 264 | 1 | 10 |
| M35 | 5113 | 9 | 3 | 3 | 685 | 1 | 264 | 1 | 10 |
| KA205 | 5418 | 9 | 3 | 3 | 1 | 1 | 1 | 4 | 10 |
| KA207 | 5689 | 97 | 3 | 240 | 1 | 1 | 1 | 45 | 10 |

CC = Clonal Complex; GJ = Gujarat; KA = Karnataka; MG = Meghalaya; MH = Maharashtra; ST = Sequence Type; TG = Telangana; UP = Uttar Pradesh.

**Supplementary Table S3**. **Details of MRSA in the study**

| **Isolate ID** | **Pulso type** | **OXA Disc** | **OXA MIC** | ***mec* A** | **SCC*mec* type** | **Toxin genes** | | | **spa type** | **ST** | **CC** |
|---|---|---|---|---|---|---|---|---|---|---|---|
| | | | | | | *hlg* | *tsst* | *pvl* | | | |
| KA10 | G | R | 32 | + | IVd | + | + | + | t3380 | 2459 | CC97 |
| KA12 | C1 | R | 16 | | | + | - | - | t4793 | 6 | CC5 |
| KA24 | C2 | R | 16 | | | + | - | - | t359 | 2459 | CC97 |
| KA26 | C1 | R | 16 | + | | + | - | + | t267 | 4992 | CC97 |
| KA54 | B2 | R | 16 | | | + | - | + | NT | 2459 | CC97 |
| KA55 | NT | R | 32 | + | | + | - | + | t359 | 2459 | CC97 |
| KA60 | C1 | R | 32 | + | IVb | - | - | + | t359 | 2459 | CC97 |
| KA62 | C1 | R | 16 | + | | + | - | + | t359 | 2459 | CC97 |
| KA69 | G | R | >64 | + | IVd | + | - | + | NT | NT | UA |
| KA70 | C3 | R | 64 | | | + | - | + | t267 | 1687 | CC97 |
| KA82 | H | R | 16 | + | | + | + | + | t359 | 2459 | CC97 |
| KA83 | H | R | 16 | + | | + | + | + | t359 | 2459 | CC97 |
| KA84 | I | R | 16 | + | V | + | + | - | t359 | 2459 | CC97 |
| KA86 | C1 | R | 64 | + | IVb | - | - | + | NT | NT | UA |
| KA94 | E8 | R | 32 | + | IVb | + | - | + | t008 | 6 | CC5 |
| KA107 | E8 | R | 16 | + | IVb | - | - | - | t10760 | 4968 | CC8 |
| KA119 | F1 | R | 32 | + | | + | + | + | NT | 672 | UA |
| KA128 | F1 | R | 32 | + | | - | - | + | NT | NT | UA |
| KA135 | E2 | R | 16 | | | + | + | + | t2700 | 2454 | CC9 |
| KA169 | C3 | R | 64 | + | | - | - | + | t359 | 4975 | CC9 |
| KA175 | E7 | R | 16 | | | + | + | + | t16344 | 2454 | CC9 |
| KA176 | E7 | R | 32 | | | + | + | + | t7867 | 9 | CC1 |
| KA193 | E7 | R | 32 | + | | + | + | - | t4522 | 2454 | CC9 |
| KA196 | D3 | R | 16 | | | + | + | + | t002 | 3081 | CC5 |
| KA200 | A1 | R | 32 | | | + | - | + | t657 | 772 | CC1 |
| KA201 | D4 | R | 16 | | | + | - | + | t7287 | 5098 | CC1 |
| MG7 | C4 | R | 32 | + | IVb | - | - | - | t7867 | 2454 | CC9 |
| MG32 | NT | R | 32 | | | - | - | - | t657 | 5419 | CC9 |
| TG14 | E4 | R | 32 | + | IVb | - | - | - | t7867 | 2454 | CC9 |
| TG15 | E2 | R | 8 | + | IVb | + | - | - | NT | 2454 | CC9 |
| UP3 | D3 | R | >64 | + | IVb | - | - | - | t7867 | 2454 | CC9 |
| UP7 | D3 | R | 64 | | | - | - | - | t17680 | 2454 | CC9 |
| UP14 | E4 | R | 64 | | | - | - | - | t7867 | 2454 | CC9 |
| UP15 | NT | R | 64 | | | - | - | - | t4522 | 2454 | CC9 |

CC = Clonal Complex; KA = Karnataka; MIC = minimum inhibitory concentration; MG = Meghalaya; NT = Non-typeable; OXA = oxacillin; ST = Sequence Type; TG = Telangana; UA = unassigned; UP = Uttar Pradesh.

## **Supplementary Table S4**. **Details of OS-MRSA in the study**

| Isolate ID | **Pulso type** | **OXA Disc** | **OXA MIC** | ***mec*A** | **SCC*mec* type** | **Toxin genes** | | | **spa type** | **ST** | **CC** |
|---|---|---|---|---|---|---|---|---|---|---|---|
| | | | | | | *hlg* | *tsst* | *pvl* | | | |
| KA48 | C2 | S | 2 | + | IVb | + | - | + | t359 | 2459 | CC97 |
| KA63 | C2 | S | 1 | + | IVb | + | - | + | t267 | 97 | CC97 |
| KA76 | G | S | 1 | + | V | + | - | + | NT | 3881 | CC1 |
| KA77 | C3 | S | 1 | + | IVb | + | - | + | t2770 | 2459 | CC97 |
| KA78 | C3 | S | 2 | + | V | + | - | + | t267 | 1687 | CC97 |
| KA81 | NT | S | 1 | + | IVb | + | - | - | t359 | 2459 | CC97 |
| KA90 | NT | S | 1 | + | IVb | + | + | + | t7683 | 2459 | CC97 |
| KA123 | F1 | S | 1 | + | - | - | - | - | t2297 | 2453 | CC97 |
| KA133 | B2 | S | 1 | + | - | + | + | + | NT | 1687 | CC97 |
| KA154 | NT | S | 1 | + | IVb | + | + | + | t021 | 1482 | CC30 |
| KA172 | C4 | S | 1 | + | - | + | - | + | t3841 | 672 | UA |
| MG3 | C4 | S | 1 | + | IVb | + | - | - | t7867 | 2454 | CC9 |
| MG5 | C4 | S | 2 | + | IVb | + | - | - | t17680 | 2454 | CC9 |
| MG8 | C4 | S | 1 | + | IVb | - | - | - | t1201 | 2459 | CC97 |
| MG9 | NT | S | 1 | + | IVb | - | - | - | t1201 | 2454 | CC9 |
| MG10 | E2 | S | 2 | + | IVb | - | - | - | t4522 | 2454 | CC9 |
| MG19 | E3 | S | 1 | + | IVb | - | - | - | t2700 | 5418 | CC9 |
| MG38 | E2 | S | 1 | + | IVb | - | - | - | t4522 | 2454 | CC9 |
| MH16 | A2 | S | 1 | + | IVb | + | + | - | t7867 | 2454 | CC9 |
| TG4 | E2 | S | 2 | + | IVb | - | - | - | t4522 | 2454 | CC9 |
| TG10 | E2 | S | 1 | + | IVb | - | - | - | t4522 | 2454 | CC9 |
| UP2 | E1 | S | 1 | + | IVb | - | - | - | t7867 | 2454 | CC9 |
| UP5 | D3 | S | 1 | + | IVb | - | - | - | t17680 | 2454 | CC9 |
| UP6 | E4 | S | 2 | + | IVb | - | + | - | t7867 | 2454 | CC9 |

CC = Clonal Complex; KA = Karnataka; MIC = minimum inhibitory concentration; MG = Meghalaya; MH = Maharashtra; NT = Non-typeable; OXA = oxacillin; ST = Sequence Type; TG = Telangana; UA = unassigned; UP = Uttar Pradesh.

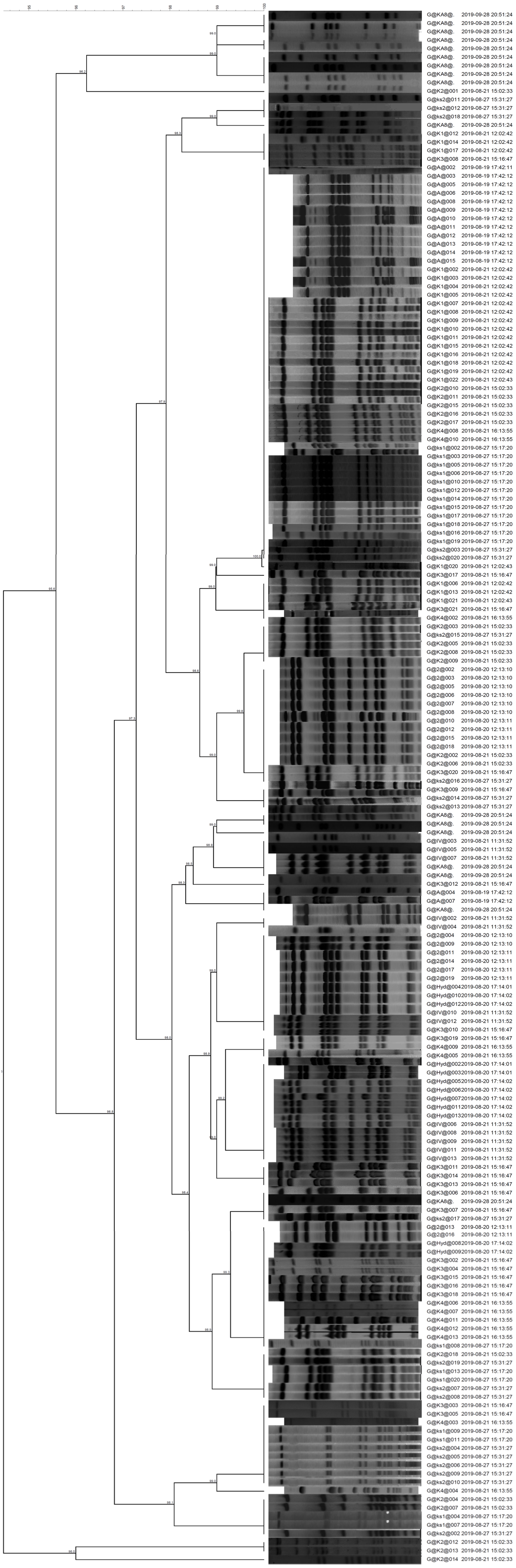


**Supplementary Figure S1. Pulsed-field gel electrophoresis data and pulsotypes analysis.** Description can be found in the Materials and Methods section.
